# There and back again: A zooarchaeological perspective on Early and Middle Bronze Age urbanism in the southern Levant

**DOI:** 10.1371/journal.pone.0227255

**Published:** 2020-03-03

**Authors:** Jane S. Gaastra, Tina L. Greenfield, Haskel J. Greenfield

**Affiliations:** 1 Institute of Arab and Islamic Studies, University of Exeter, Exeter, United Kingdom; 2 St. Thomas More College, University of Saskatchewan, Saskatoon, Saskatchewan, Canada; 3 Department of Anthropology and St. Paul’s College, University of Manitoba, Winnipeg, Manitoba, Canada; University at Buffalo - The State University of New York, UNITED STATES

## Abstract

Multiple arguments for or against the presence of ‘urban’ settlements in the Early Bronze Age of the southern Levant have identified the need to compare these settlements against their rural hinterlands through multiple lines of evidence. This meta-analysis of zooarchaeological data from the region compares and identifies patterns of animal production, provisioning and consumption between the supposed “urban” and rural sites of the southern Levant from the Early Bronze (EB) against the (more widely recognised urban) Middle Bronze (MB) Ages. It also identifies distinct and regionally specific patterns in animal production and consumption that can be detected between urban and rural sites of the southern Levant. The taxonomic and age profiles from EB Ia and Ib sites do not demonstrate any urban versus rural differentiation patterning, even though fortifications appear in the EB Ib. Beginning in the EB II and clearly visible in the EB III, there is differentiation between rural and urban sites in the taxonomic and age proportions. Differentiation is repeated in the MB II. The clear differentiation between “urban” and rural zooarchaeological assemblages from the EB II-III and MB suggest that rural sites are provisioning the larger fortified settlements. This pattern indicates that these sites are indeed urban in nature, and these societies are organized at the state-level. From the EB II onwards, there is a clear bias in the large centres towards the consumption of cattle and of subadult sheep and goats with a corresponding bias in smaller rural sites towards the consumption of adult sheep and goats and a reduced presence of cattle. After the emergence of this differential pattern, it disappears with the decline in social complexity at the end of the Early Bronze Age, only to come ‘back again’ with the re-emergence of urban settlement systems in the Middle Bronze Age.

## Introduction

The origins and nature of complex societies in the southern Levant have seen extensive debate over the last several decades. The southern Levant (the territories of modern Israel, Jordan, and Palestinian Authority) is one of the earliest areas outside of the regions with primary state-formation (i.e. Mesopotamia and Egypt) to begin to exhibit many of the characteristics of early or archaic complex societies. Large fortified settlements appear and dominate multiple tier settlement system hierarchies across the region during the Early Bronze Age (**EB** hereafter). The presence of these large fortified sites is presumed to be urban in nature and by implication signal the emergence of state-level societies in the region ([1], [2]; [3]; [4]). These secondary state-level urban societies develop several hundred years after the appearance of comparable societies Mesopotamia during the later Chalcolithic ([5]; [6]; [7]; [8]).

Yet, the nature of southern Levantine EB settlements and associated societal organisation continues to be debated. Views of this period, and the presence or absence of ‘urban’ state-level societal structures, vary greatly between researchers. Supporters of EB urban state-level societies marshal evidence from both the largest urban to the smallest village sites in the EB ([9]; [10]; [11]; [12]; [3]; [13]; [14]). At the other end of this debate is the complete rejection of an urban state-level character for even the largest EB sites ([15]; [16]; [17]; [18]). Essentially, scholars have argued that urbanism and state-formation appear in this region only during the Middle Bronze Age (**MB** hereafter) or that they are present in both the EB and MB as in Mesopotamia and the northern Levant ([19]; [20]; [21]).

Given the disagreements over interpretation, there is a clear need to comparatively consider multiple aspects of social and societal development in order to understand the nature of EB society in the southern Levant. This has become apparent within both viewpoints ([15]; [22]; [3]; [6]). Recent investigations of putative urbanism in this region have successfully utilised comparisons between EB and MB settlement dynamics and productive specialisation ([3]) between developments in the southern Levant with those in the northern Levant and Mesopotamia ([3]; [23]), and also between regions within the southern Levant ([24]; [25]). Recent models of early state formation have identified three key features in their evolution: social stratification, central governance and economic specialisation ([26]; [27]). Underlying all models of early state and urban formation is the assumption of the production of surplus, particularly food, which releases part of society for non-food producing tasks ([28]; [29]; [30]; [31]; [32]; [33]; [34]). Domestic livestock are one of the key economic foundations for surplus production that is at the essence of any theory for the emergence of complex societies—including state-level societies and urban centres ([35]; [36]; [37]; [38]; [39], [40], [41]).

As a contribution to the ongoing debate as to the nature of EB settlement and society (state-level urban or otherwise) in the southern Levant, this paper conducts a meta-analysis of zooarchaeological evidence for animal production and site provisioning from the later Chalcolithic to MB between sites within the southern Levant. We begin our study in the later Chalcolithic to establish a base-line for what most scholars recognise as both pre-urban and pre-state societies in this region. The study continues into the MB, which is widely characterised as having state-level urban societies ([42]; [43]). Both EB and MB sites that have been characterised as ‘urban’ on the basis of multiple lines of evidence (e.g. architecture, material culture, size, fortification, etc.) will be included in this study to highlight and address their functional relationships with rural sites in their hinterland—specifically that of the nature of animal food (local) production and/or (non-local) provisioning (see [44]). This comparative assessment will allow for the comparison of patterns and relationships in animal production between identified rural and urban sites during both the contested state-level urbanism of the EB with the largely un-contested state-level urbanism of the MB.

## Defining urbanism: A comparative approach to southern Levantine Early Bronze Age states and urbanism

In recent years, there has been a growing recognition that models based upon Mesopotamia or Egypt are not directly applicable to the secondary states in the neighbouring regions such as the southern Levant where the scale and nature of urban and complex societies are different ([45]; [17]; [46]; [3], [47]; [48]; [49]). It is clear that developments in each region cannot be forced into a single theoretical framework. Further, there are recognisable regional differences in the evolution of complex societies between the northern and southern Levant ([19]; [18], [50]). The presence of state-level urban centres in the southern Levant has been subject to extensive debate in recent decades. Some scholars view the regional development of complex societies in the southern Levant as increasingly integrated into regional kingdoms ([51], [2]), others as a series of independent city-states ([3], [52]), while some view it from the perspective of the non-state corporate village ([45]; [17]).

Given the contemporaneous presence of urban centres in nearby regions (namely Mesopotamia), discussions of urbanism in the southern Levant have often involved comparisons of ‘urban’ sites and site function between these regions ([15]; [6]; [53]). Yet, the comparison is not a simple one since the size, scale, and nature of urban centres can be vastly different. Territorial extents, settlement hierarchies, settlement size, public architecture, mortuary complexity, etc. are all much smaller in absolute size and scale in the southern Levant ([3], [52]). The development of fortified large settlements in this region during the EB have been dated to the later stages of the EB Ib period (see Table 1) and continue through the EB II into the earlier phases of the EB III ([54]; [53]). The presence of large numbers of fortified settlements throughout the southern Levant beginning in the EB Ib is well attested, although the wide range of sizes for these fortified settlements (from <1ha up to 25ha) suggests that the presence of fortifications alone is not a useful identifier of urbanism ([15]; [44]). There is no evidence of written sources from southern Levantine sites during the EB, indicating that writing was either not in use or incorporated a medium which has not survived ([55]). Administrative and ritual complexes are generally limited to the largest sites in this region ([15]; [56]).

**Table 1:** Chronological periods used in this study. Based on: [58]; [110] and [111].

Phase	cal BCE	Period
1	4400	Ghassulian/Beershevan
	4300	
	4200	
	4100	
	4000	
	3900	
	3800	
	3700	
2	3600	EB Ia
	3500	
	3400	
3	3300	EB Ib
	3200	
	3100	
	3000	
4	2900	EB II
	2800	
5	2700	EB III
	2600	
6	2500	EB IV
	2400	
	2300	
	2200	
	2100	
7	2000	MB I
8	1900	MB II
	1800	
	1700	

The large, fortified and planned part of the regional settlement system in the southern Levant experienced changes at the end of the EB III period. In what has been termed a ‘collapse’ ([57]; [58]; [59], [60]). These “urban” settlements are largely abandoned at the end of the EB III and do not re-emerge until the subsequent MB II period. In the northern Levant and Mesopotamia, a ‘collapse’ is also seen (sometimes violently so) somewhat later in the final century or two of the third millennium BCE ([13]; [61]; [6]; [62]; [53]). While political collapse combined with environmental change are considered the likely causes of the loss of the ‘urban’ peak of the settlement system in the southern Levant at the end of the EB III, similar changes in the northern Levant and Mesopotamia have been widely considered linked to the ‘4.2 kya climatic event’ period of aridity at the end of the third millennium BCE ([63]; [64], [62]). This association is not so clear in the southern Levant ([65]; [66]; [67]; [61]; [68]). After this loss of state-level urban structures at the end of the EB, urban states come ‘back again’ in all three regions in the succeeding Middle Bronze Age. Large (>50ha), fortified settlements with administrative centres and evidence for the control of craft production and trade are widely agreed to be present across both Mesopotamia and the southern Levant during this period (e.g. [69]; [5]; [15]; [70]; [71]; [72]; [6]; [73]).

### Does size matter?

In comparison with urban sites from Mesopotamia which reach size of c.100 ha during the EB ([69]; [5]; [6]) or MB urban sites in the southern Levant which reach up to 80 ha (e.g. Hazor—[70]), the top of the settlement hierarchy of EB southern Levantine sites are argued by some to be too small to represent a state-level tiered settlement hierarchy with an urban character ([15]). Yet, recent regional surveys of EB sites in the southern Levant provide a wealth of support for a tiered settlement hierarchy ([74]; [75], [76]). Other scholars (following [44]) dispute the utility of directly comparing urbanism between regions on the basis of overall settlement size rather than the function of these large, planned sites within regional settlement networks ([15]; [77]; [78]; [79]). This approach focuses on urbanism as a cluster of attributes rather than the presence of a defining criterion such as size or the presence of walls ([44]; [80]). Urban sites are here considered as “a permanent settlement within the larger territory occupied by a society considered home by a significant number of residents whose activities, roles, practices, experiences, identities, and attitudes differ significantly from those of other members of the society who identify most closely with “rural” lands outside such settlements.” ([44], pp.526). It is through comprehensive analysis of settlement system structure and functional relationship with local rural sites that urban sites are identified rather than via a set list of individual or ‘universal’ urban factors.

## Urban zooarchaeology

Few specific zooarchaeological comparisons of urban settlement function have been made for the EB or MB southern Levant (although see [25]; [81]). However, a significant body of zooarchaeological research into comparative differences in urban provisioning and access to animal resources exists for contemporaneous sites in Mesopotamia as well as for later periods of the southern Levant (e.g. [81]; [82]; [83]). These studies have identified common trends in faunal data which can be used to determine the both presence of elites as well as more specifically the identification of functional relationships between urban vs. rural sites.

### Status and political economy

One of the core components behind distinguishing between urban and rural ends of the settlement and distribution systems is the identification of unequal access to resources between ‘elite’ and ‘non-elite’ segments within past societies. More elites typically reside in upper tier settlements. The political economy or control of resources can be identified in differences in the type and quantity of trade and craft goods, residential structures, mortuary elaboration, and many other constructs ([26]; [5]; [44]; [84]; [78]; [73]; [40]). Differences in access to resources between these groups are not universal but can be expressed in a panoply of ways depending on each society. Thus, in zooarchaeology as in archaeology more generally, there are no universal determinants of either ‘elite’ or ‘urban’ consumption ([85]). Rather, it is through comparative analyses of the available data that repeated trends are identified both in individual cultures and more general cross-cultural patterns. Zooarchaeologically, proportional representation of different animal taxa, the age structures of animals recovered from different contexts (e.g. elite/non-elite or urban/rural), and the portions of the body consumed are the most common comparative data used to identify differential access to resources by a particular segment of society, or the control of resources and their distribution across particular settlement contexts. While the specific species involved varies across time and space, elite consumption is most commonly identified through greater access to higher-status animals, whether these be a particular domesticate (e.g. cattle, pigs) or a greater proportion and range of exotic or wild animals through increased leisure time for hunting or trade in preserved (or live) foodstuffs or disproportionate access to meatier portions of the animal carcass ([86]; [87]; [85]; [84], [88]; [25]; [89]; [82]; [83]; [90]; [39], [40], [41].

### Commoners, elites and urbanites

The identification of status differences between sites or areas of sites does not, in and of itself, indicate the presence of urban settlements. While elites are more commonly found on top-tier settlements within state- or chiefdom-level societies, this does not necessarily discriminate the presence of urban sites. If we are examining the presence of urban settlements, this pre-supposes both the existence of elites as well as (more importantly) a city-state or regional-state level of organisation within the region and time period under consideration. Thus, it is important to determine whether a given settlement exerted control over an array of satellite settlements to the extent that it exhibits differences in function from that of the smaller settlements under its control (e.g. [44], see above). The important discriminatory factor in the identification of urban sites is then in their function in contrast to non-urban sites, in their control over a surrounding landscape of satellite sites and resources produced by and traded through this landscape ([44]; [91]). The control of resources by urban settlements can be seen both through comparisons of material culture as well as through the economic provisioning of urban vs. rural settlements. In zooarchaeological research this is seen through repeated differences in access to and/or control of animal resources ([86]; [87]; [85]; [92]; [84]; [93], [94]; [40], [41]).

There has long been an identified difference between rural food-producing sites and urban food-consuming sites of the EB and MB of both northern and southern Mesopotamia as well as later periods of the southern Levant. The urban or rural function of settlements has been identified through a number of differences in the fauna recovered from these groups of sites. Some of these are indicators of status, such as increased differential access to particular species or an increased range of species (e.g. [95]; [96]; [84]; [97]; [98]; [99]; [39], [40], [100], [101], [41], see above). Beyond indicators of status, functional differences between rural and urban sites can be seen in the age ranges of slaughtered livestock recovered from sites (e.g. for Mesopotamia: [84]; [98]; [102]; [90]; [99]; [100]; [103]; for post-MB periods of the southern Levant: [104]; [81]; [83]). Urban sites in both cases have been found to demonstrate an over-abundance of ‘prime-aged’ (subadult or younger) domesticates which are correspondingly lacking in rural sites ([105]). This has been widely accepted as indicating the provisioning of food to urban-consumer sites from rural-producer sites and provides a clear indication functional differences between urban-consumer sites beyond the presence of elites (e.g. [106]; [104]; [102]; [90]; [40], [41]). It is through the identification of these repeated patterns of urban-rural contrast that we are able to confidently identify the existence of urban provisioning systems and their associated political economy in a given region and chronological period ([86]; [85]; [84]).

### Southern Levantine urban zooarchaeology

It would be overly simplistic to assume that the specific patterns of urban comsumption identified for contemporaneous urban sites in Mesopotamia would be replicated in the southern Levant. For the purposes of this study, what is noteworthy about the above studies of urban zooarchaeological provisioning are the repeated demonstration of differences between urban and rural sites. The increased frequency of pigs/hunted animals, or of prime subadult sheep and goats, is not expected to be identical in the EB or MB southern Levant due to differences in environment and cultural values. However, based upon the studies cited above, we can expect that urban and rural sites of the MB southern Levant would exhibit differences in taxonomic distributions and age profiles from contemporaneous rural sites. Similarly, if urban sites were indeed present in the southern Levant during the EB, we would expect such a pattern to be apparent between the ‘urban’ and rural sites.

Given current debates over the status of ‘urban’ sites in the EB of the southern Levant, it is timely to consider the issue from a different perspective—the functional relationships between urban vs. rural sites as seen through their economic provisioning of and how these change over time. This study will conduct a comprehensive comparative assessment of zooarchaeological data from the region determine the relationship between the putative urban and rural sites. An earlier attempt was made, although it was limited by the inadequate chronological controls and geographic representation of faunal data available at the time ([24]). While multiple studies have subsequently been made between individual sites or regions ([97]; [107], [108]; [25]), there has not been a broader comparative assessment made between urban and non-urban patterns of provisioning via the examination of animal resource management for the EB and MB.

## Data and methods

### Some time and space considerations

The southern Levant is not a uniform homogeneous region. It is characterized by extreme environmental (with climatic and topographic) diversity and complexity. There are differences in cultural development between its eastern, western, southern, and northern regions ([17]) which may be partly due to environmental differences. Given these factors, we expect that there will be a diversity of animal exploitation strategies reflecting local adaptations ([109]). However, if the need to provision state-level urban centres overrode that of local adaptations, we may expect to see more regionally uniform patterns in domestic livestock exploitation. Hence, in order to be able to evaluate larger-scale patterns of animal exploitation, assemblages from diverse ecological settings across the region must be considered. Only meta-analysis has this capability.

**Fig 1:**
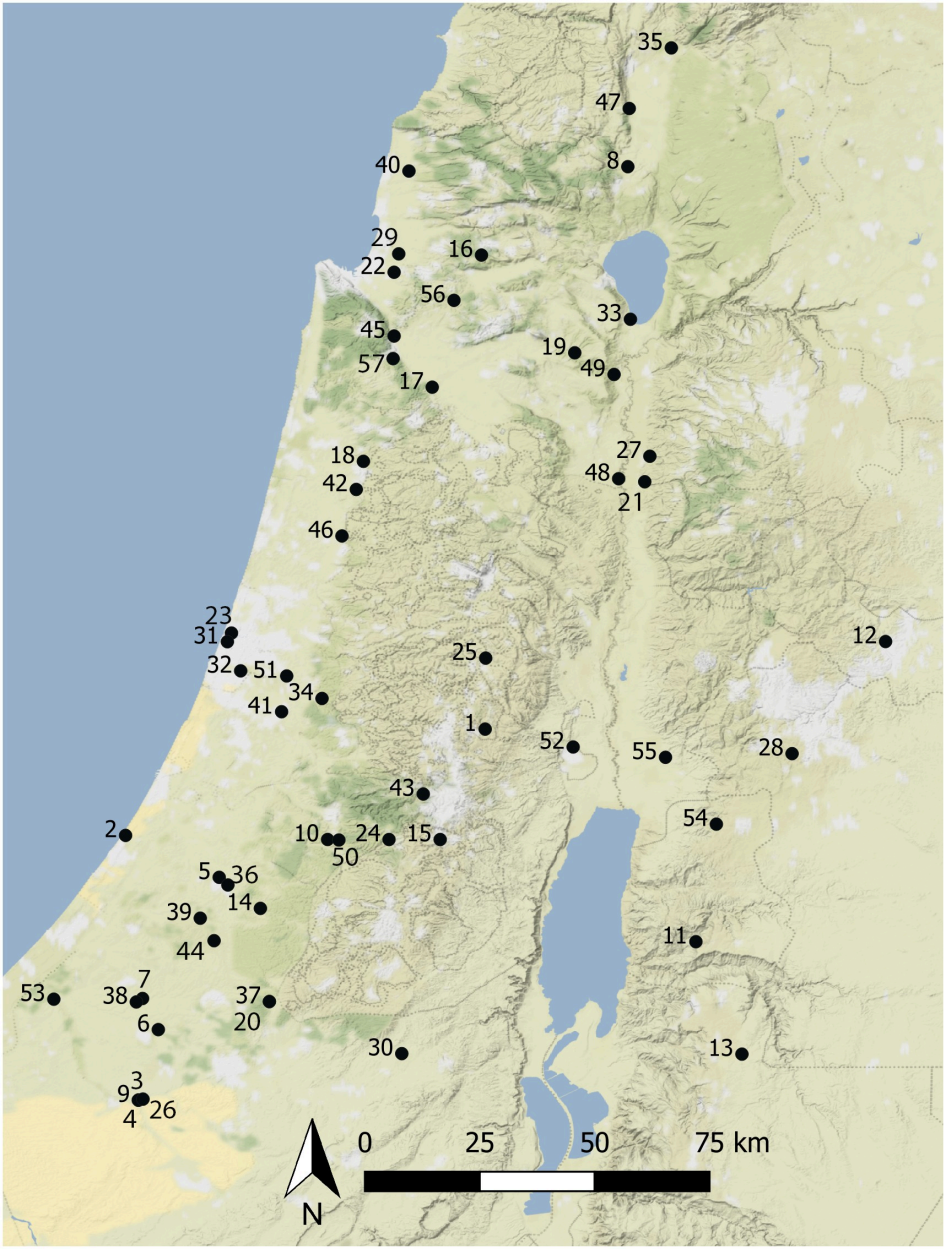
Location of the sites used in this study. Further details on these sites are available in Table 2. Basemap: Stamen Terrain (obtained through QuickMapServices QGIS plugin), (cc) OpenStreetMap contributors.

Chronologically, our study begins with the later Chalcolithic (Table 1. There are 17 site samples from the late Chalcolithic (c.4500-3600 cal BCE), 9 samples in the EB Ia (c.3600-3300 cal BCE), 14 from the EB Ib (c.3300-2900 cal BCE), 13 from the EB II (c.2900-2700 cal BCE), 12 from the EB III (c.2700-2500 cal BCE), 4 from the EB IV (c.2500-2000 cal BCE), 2 from the MB I (c.2000-1900 cal BCE) and 19 from the MB II (c.1900-1700 cal BCE). This dataset represents all published zooarchaeological data from the above periods of the southern Levant which could be located by the authors and which meets the criteria for selection within this study (see below and Fig 1). In total it comprises 89,241 elements from 91 phases at 57 sites. Faunal samples from this dataset provided additionally 48 ovicaprine dental age samples from 31 sites. Chronological phases used here for the Early Bronze Age follow the chronologies of Regev et al. ([110]) and Hölfmayer ([58]; [111]). Where sites and site samples have been ascribed to a specific relative phase within the EB or MB (e.g. EB III or MB II) this phasing has been retained for the present analysis. Samples for which 14C dates are available (e.g. Ai et-Tell EB Ic/EB II) but for which the specific period identification is less certain have had their phasing decided on the basis of radiocarbon dates. Sites which contained faunal samples both for individual phases (e.g. EB II) as well as samples spanning multiple phases (e.g. EB II/III) have contributed only those phases assigned to a single specific phase. For sites where faunal samples are not assigned to a specific chronological phase (e.g. Jericho—[112]) but have been combined to span multiple phases (e.g. EB II/III or MBA) have been omitted from this analysis. Where multiple samples were present for the same site within the same chronological phase, these were combined to form a single phase-sample. This was not done, however, when faunal samples came from different context types within the same site (e.g. urban, lower town of urban or cultic). Within each period, sites are divided between urban and rural with a spectrum of six classifications to allow for levels of indeterminacy in the ‘urban’ or ‘rural’ status of excavated sites (see below and Table 2).

**Table 2:** Summary information for faunal samples compared in this study. The date ranges for chronological phase-groups and periods can be found in Table 1. Full bibliographic citations for the faunal samples used in this study can be found in SI3. Those sites which provided dental age data are marked in the ‘Age’ column with a ⋆.

No.	Site	Phase	NISP	Age	Status	Reference
1	Ai et-Tell	3	247	⋆	?urban	Hesse & Wapnish 2001
		4	601	⋆	urban	Hesse & Wapnish 2001
		5	118		urban	Hesse & Wapnish 2001
2	Ashquelon Afridar	2	11306	⋆	rural	Kansa 2004; Whichiter 1999; Sade in Golani 2008
3	Bir Abu Matar	1	257		rural	Josien 1955
4	Bir es-Safadi	1	532		rural	Josien 1955
5	Gat Govrin	1	210	⋆	rural	Ducos 1968
6	Gilat	1	1065	⋆	cultic	Grigson 2006
		2	1657		cultic	Grigson 2006
7	Grar	1	1279	⋆	rural	Grigson 1995
8	Hazor	8	2410		cultic	Marom et al 2017
		8	2512		urban	Marom et al 2017
9	Ḥorvat Beter	1	228		rural	Grigson 1993
10	Horvat Illin Tahtit	3	1954	⋆	rural	Allentuck 2013
11	Khirbat Iskander	6	330		indet.	Metzger 2010
12	Khirbet al-Batrāwī	5	533	⋆	urban	Alhaique 2008, 2012
		5	124		lower	Alhaique 2008, 2012
		6	402	⋆	rural	Alhaique 2008, 2012
13	Khirbet el-Minsahlat	6	553	⋆	?rural	Makarewicz 2005
14	Lachish	7	2514	⋆	indet.	Croft 2004
		7	1318		cultic	Croft 2004
		8	1258	⋆	urban	Croft 2004
15	Manahat	8	686	⋆	rural	Horwitz 1998
16	Marj Rabba	1	6234	⋆	rural	Hill et al 2016: Price et al 2013
17	Megiddo	3	2960	⋆	cultic	Hesse & Wapnish 2001
		5	673	⋆	cultic	Wapnish & Hesse 2001
18	Metzer	1	345	⋆	rural	Ducos 1968
19	Munhatta	1	358	⋆	rural	Ducos 1968
20	Nahal Tillah	2	318	⋆	rural	Grigson in Wichiter 1999; Kansa et al. 2006
		3	1305	⋆	rural	Grigson in Wichiter 1999; Kansa et al 2006
21	Pella (Tabaqa Fahl)	1	949		rural	Mairs in Bourke et al 1994, 1998
		4	1187		urban	Mairs in Bourke et al 1994, 1998
		8	3554		urban	Mairs in Bourke et al 1994, 1998
22	Qiryat Ata	3	237		rural	Agha 2014; Horwitz 2003a, 2013; Sade 2000
		3	373		?rural	Horwitz 2003a, 2013; Sade 2000
		4	810		?urban	Agha 2014; Horwitz 2003a, 2013; Maher 2014a
23	Ramat Aviv Hotel	8	230		rural	Sade 2006
24	Refaim Valley	6	308		rural	Horwitz 1989
		8	254		rural	Horwitz 1989
25	Shiloh	8	651	⋆	rural	Hellwing et al 1993
26	Shiqmim	1	4159	⋆	rural	Grigson 1987; Levy et al 1991, 1993; Wichiter 1999
27	Tall al-Ḥuşn	3	1087		rural	Mairs in Bourke et al 1998
28	Tall al-’Umayri	3	568	⋆	?rural	Peters et al 2002
		5	8587	⋆	?rural	Peters et al 2002
		6	107		rural	Peters et al 2002
29	Tel Aphek	3	128		rural	Hellwing 2000
		4	120		?urban	Hellwing 2000
		8	1095		urban	Hellwing 2000
30	Tel Arad	3	383		rural	Lernau 1978
		4	1097	⋆	urban	Lernau 1978
		5	311		rural	Lernau 1978
31	Tel Aviv	1	567	⋆	rural	Ducos 1968
32	Tel Azor	2	229		rural	Horwitz in Golani et al 1999
33	Tel Bet Yerah	2	122		rural	Berger 2018
		3	326		?rural	Berger 2018; Cope 2006
		4	878		?urban	Berger 2018; Cope 2006
		5	591		urban	Berger 2018; Cope 2006
34	Tel Dalit	3	225	⋆	rural	Horwitz et al. 1996
		4	813	⋆	?urban	Horwitz et al. 1996
35	Tel Dan	5	132	⋆	urban	Wapnish & Hesse 1991
36	Tel Erani	3	544		urban	Wojtal et al. 2016
37	Tell Halif	5	1972	⋆	indet.	Zeder in Seger et al 1990
38	Tel Haror	8	5183	⋆	urban	Klenck 1996, 2002
		8	1019	⋆	cultic	Klenck 1996, 2002
39	Tel el-Hesi	5	907	⋆	urban	Peck-Janssen 2006
40	Tel Kabri	2	137		rural	Horwitz 2002
		8	836	⋆	urban	Marom et al 2015
41	Tel Lod	2	466	⋆	indet.	Horwitz in van den Brink et al 2015
42	Tel Magal	4	112		indet.	Kehati 2017
43	Tel Moza	2	185		rural	Sade 2009
		7	246		rural	Sade 2009
44	Tel Nagila	8	485	⋆	indet.	Ducos 1968
45	Tel Qashish	4	290		?urban	Horwitz 2003b
		5	233		?urban	Horwitz 2003b
		8	235		?urban	Horwitz 2003b
46	Tel Te’enim	5	100		rural	Horwitz 2011
47	Tel Te’o	1	163		rural	Horwitz 2001
48	Tel Tsaf	1	118		rural	Hellwing 1988; Hill 2011
49	Tel Yaqush	3	503	⋆	rural	Hesse & Wapnish 2001
		4	393	⋆	rural	Hesse & Wapnish 2001
		5	346	⋆	indet.	Hesse & Wapnish 2001
50	Tel Yarmut	4	218	⋆	?urban	Davis 1988
		5	965	⋆	?urban	Davis 1988
51	Tel Yehud	8	108		indet.	Horwitz in van den Brink et al 2014
52	Tell el-Mafjer	1	1026		rural	Al-Zawahra 2008
53	Tell Jemmeh	8	132		indet.	Maher 2014b
54	Tell Madaba	3	441		urban	Griffith 2016
55	Tulaylat al-Ghassul	1	1108		rural	Mairs in Bourke et al 1995; Bourke et al 2000
		2	1412		rural	Mairs in Bourke et al 1995; Bourke et al 2000
56	Yifta ḥ’el	2	283		rural	Horwitz 1997
57	Yoqne’am	8	815	⋆	?urban	Horwitz et al 2005

### Taxonomic and age distributions

Data for taxonomic comparisons are restricted to taxonomic abundance by Number of Identified Specimens (NISP) as the common type of zooarchaeological data consistently reported across the dataset. Only counts of specimens identifiable to species or genus have been included in the present analysis with a few minor exceptions (see below)—identifications to broader categories such as ‘small ruminant’ or ‘large mammal’ have been removed from comparisons. Occasional reporting (1 sample from Khirbet al- Batrāwī) of *Sus sp*. (indeterminate wild or domestic pig) has been allocated pro rata based upon the proportions of domestic vs. wild pig in each assemblage ([109]; [113]). Other identifications of *Sus sp*. or *Equus sp*. have been assigned to wild or domestic following the descriptions of the analyst as given in each faunal publication (e.g. Tall al-‘Umayri) This has been done as the best means by which to account for the occasional use of indeterminate wild or domestic *Equus sp*. and *Sus sp*. by faunal analysts. The original counts and their pro-rata allocation can be seen in the faunal dataset SI1. *Ovis/Capra* (sheep/goat) have not been subjected to pro-rata allocation as difficulties in distinguishing between these species in zooarchaeological identification commonly results in the majority of identifications belonging only to this combined group. Additionally, separate counts of individually identified *Ovis* and *Capra* are not always reported by analysts (from the 91 samples 30 did not report individual *Ovis* and *Capra* counts). Therefore, all identified *Ovis aries* and *Capra hircus* specimens reported by analysts have been subsumed here within the larger taxonomic category *Ovis/Capra*, which is common across all analysts.

Data for comparisons of age profiles for sheep and goats were restricted to dental age data following Payne ([114]) age groups. This has been done as dental age profiles provide an estimated age for each animal on the basis of dental eruption and wear as opposed to comparative ages (younger than or older than) obtainable from epiphyseal fusion ([90]). These dental age data have the additional benefit of providing a standard reporting format for researchers and thus allows more detailed comparisons of age profiles between site samples than the more general age categories of ‘infant’, ‘juvenile’, ‘sub-adult’ or ‘adult’. *Ovis* and *Capra* dental data used in age profile reconstructions are primarily published only as *Ovis/Capra*. While the exploitation of these species would and should be different given that their secondary products are different ([115], [116]; [117]), the limitation of age profile data only to sites for which separate information is available for *Ovis* and *Capra* would have required the majority (40 out of 46 samples) of sites to be deleted from the analysis. In order to mitigate somewhat this necessary combination of *Ovis* and *Capra* dental samples, samples have been categorised according to the proportions of *Ovis* and *Capra* recorded in the overall sample NISP. For those where individual *Ovis* and *Capra* dental data has been published, these have been included separately, For those sites where such data is not available, age profiles have been grouped according the dominance of *Ovis* or *Capra* within the faunal sample, as can be seen in the dental age dataset, SI2. Dental age data for cattle was not included in this study due to the low frequencies of this taxon across the majority of samples in the dataset (resulting in smaller ageable samples) and the sparse reporting of dental age data for cattle by analysts (cattle dental data was available for only 7 out of 91 faunal samples).

Wild taxa were combined into functional taxonomic groups for comparison. This was done so as to eliminate as much as possible differences in the distribution of individual species. As the taxonomic category of ‘large game’ encompasses all genera of deer this category is also able to include the somewhat broader taxonomic category of ‘cervidae’ occasionally reported by analysts. Similarly, the taxonomic category of ‘small game’ encompasses all wild taxa larger than a hedgehog and smaller than a wolf and thus able to include the broader taxonomic category of ‘small carnivore’ occasionally reported by analysts. For this same reason, the taxonomic category ‘large carnivore’ includes the occasionally-used taxonomic category of ‘large carnivore’. All identified mammals larger than a hedgehog (macro-mammals) have been included in the present dataset. Additional taxonomic groups such as birds, fish and microfauna were excluded from the dataset as these are more strongly affected by variations in recovery practices and reporting by individual analysts ([118]). A minimum NISP cut-off of 100 taxonomically identified macro-mammals was applied for each phase-sample following the methods of Gaastra and Vander Linden ([113]). Correspondence analysis was conducted on NISP counts of taxa following these restrictions.

### Distinguishing between urban and rural and upper and lower towns

The identification of urban sites in the archaeological record is not straightforward. In many sites such determination is tricky as the identification of the size and organisation of settlements is dependent on the size and extent of excavation areas to determine the presence of settlement plan, fortifications and/or public buildings. This is particularly a problem for sites which were occupied in both the EB (or MB) as well as later periods, as subsequent occupational deposits may overlie earlier occupation areas making large areas of exposure more difficult. Given the debates as to the size of sites in the EB vs. those of the MB and sites of Mesopotamia, there are some additional considerations—the size of sites as reported by excavators varies in the consideration of the acropolis area only (walled area or tell summit) or the inclusion of the lower town (e.g. [119]; [22]; [120]). Likewise, the largest urban sites of Mesopotamia extend over not only one acropolis and lower town but in many instances cover the summits of multiple hills which (e.g. [6]), the identification of such extensive settlements therefore requires extensive survey and off-tell excavation to determine the size of settlements in any one period, and the true extent of lower town areas may be difficult to establish. In the identification of sites as urban, this study has taken these difficulties into consideration by dividing faunal site samples into a range of categories. Sites identified as ‘urban’ are those demonstrating planned settlement with fortifications and public/monumental architecture, as well as having an estimated size at the upper end of sites for the given period (e.g. c.8 hectares or greater for the EB) as well as an identification as ‘urban’ by the excavators. A second category of urban site used here is ‘?urban’ to designate sites which exhibit a planned and fortified occupation but where the presence of public architecture is not certain nor is the existence of a large area of settlement. Sites which possibly exhibit planned and fortified settlement are listed as ‘indeterminate’. Those which exhibit only one of the three criteria (planned, fortified, public architecture) and which do not demonstrate an extensive area of settlement are categorised as ‘?rural’ and those which are known to have been unplanned, unfortified and without public/monumental architecture are categorized as ‘rural’. In addition, sites which functioned as local cultic centres or cultic complex areas from within sites are classified as ‘cultic’ as any ritual manipulation of animals within such precincts may alter their faunal profile relative to the remainder of the site or relative to similar sites in the vicinity.

Some sites have both an upper and lower tell/town. Some putative ‘urban’ settlements were excavated in both upper and lower town areas without a clear division of faunal samples reported by zooarchaeologists. It is therefore not always clear whether these pooled samples may mask differences between upper and lower town contexts or whether the dominant portion of some faunal samples (e.g. Ai et-Tell and Tel Dan, see Table 2) derive from excavations on the acropolis or from those in the lower town. Where excavations have taken place in lower town areas, and these have been considered separately by zooarchaeologists, these have been designated as ‘lower town’ (Khirbet al-Batrāwī). Where the locational context of samples is indeterminate, these lumped samples have been classified at the site level and compared with samples from known acropolis/lower town samples.

### Analysis techniques

The data were analysed using a combination of statistical methods, all undertaken in R ([121]). Correspondence analysis was employed to compare combined variations in both the representation of all taxa between regions and phases ([122]). Comparisons of proportions of individual taxa in assemblages was made via jitter charts using the R package ‘ggplot2’ ([123]). Ternary diagrams of dental age groups for ovicaprines were made using the R package ‘ggtern’ ([124]). ANOVA calculations were made using the R package ‘stats’ ([121]).

## Observations

### Taxonomic proportions

**Fig 2:**
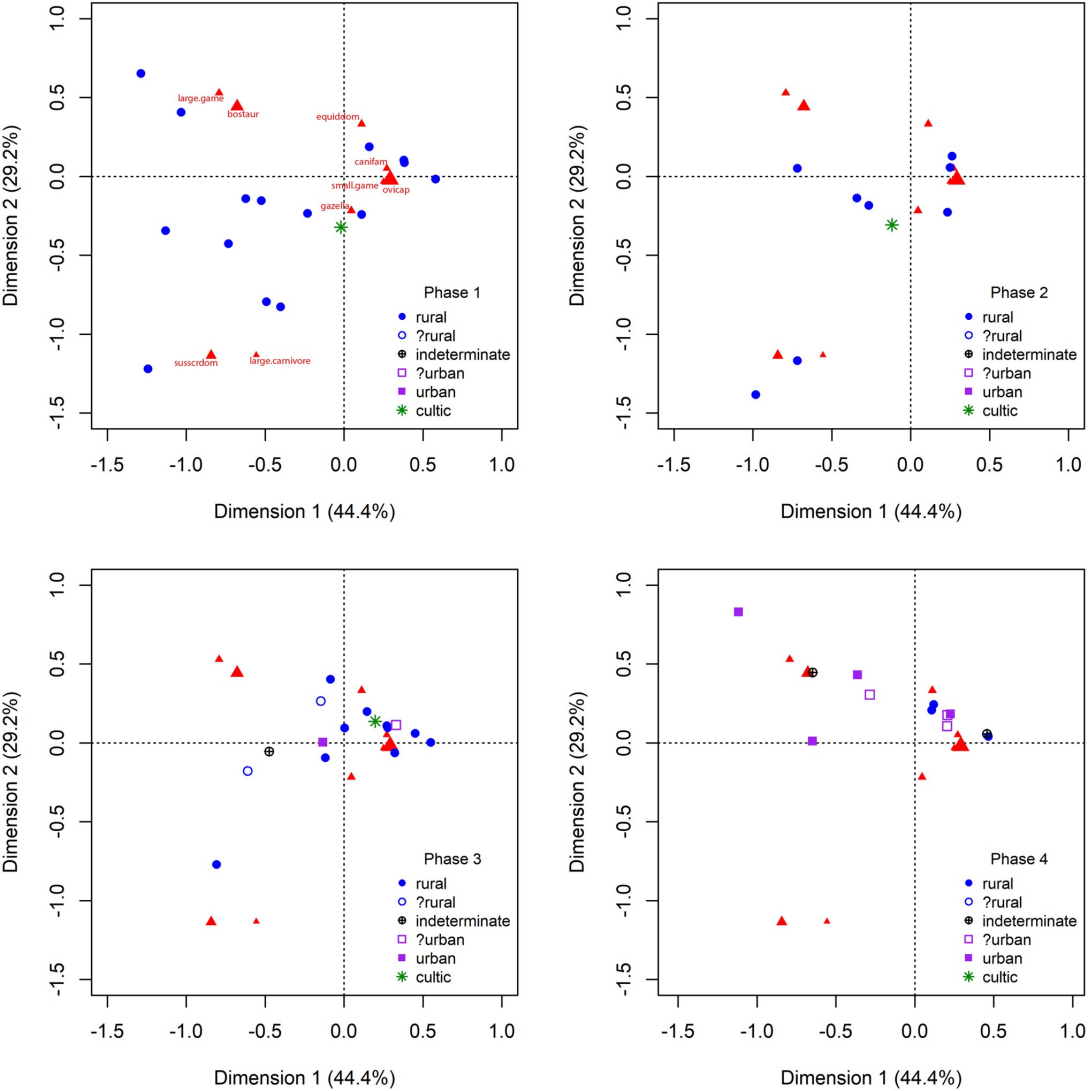
Correspondence analyses of taxonomic distributions for Phases 1 through 4.

**Fig 3:**
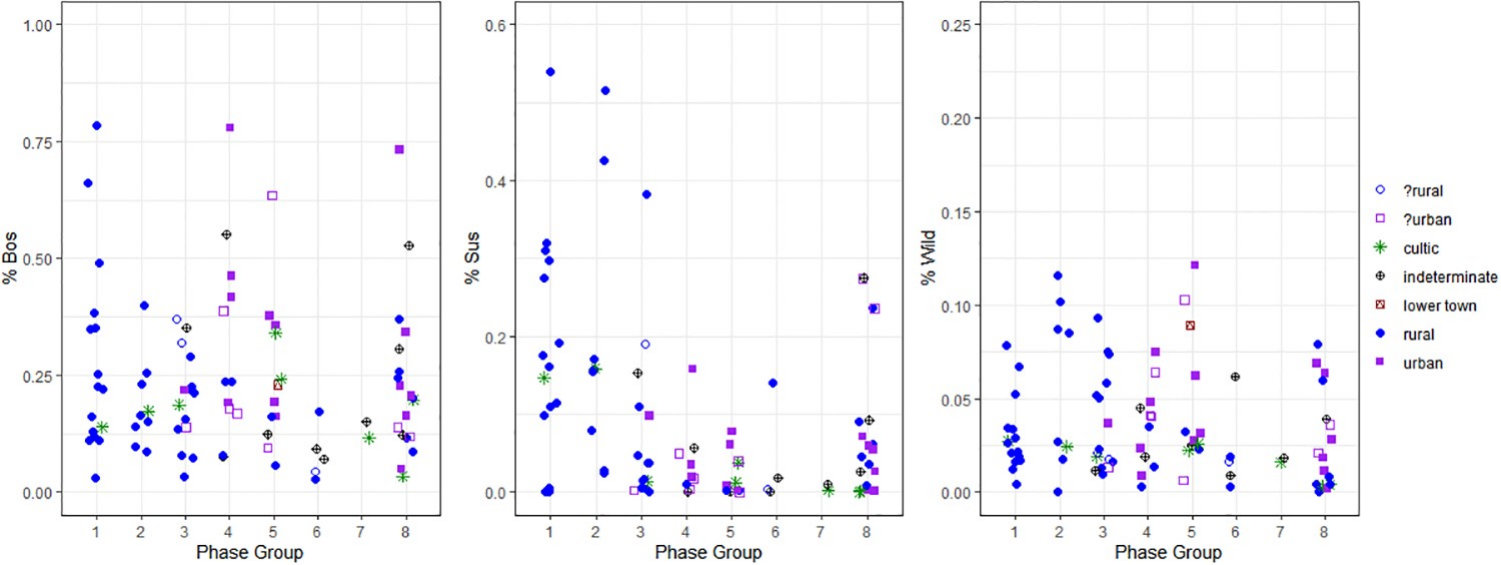
Stripcharts indicating the proportional representation of wild taxa as well as of cattle and pigs within domesticates for each phase.

Correspondence analyses conducted on taxonomic proportions show no patterning during the later Chalcolithic (Phase 1: c.4400-3600 cal BCE) and EB1a (Phase 2: c.3600-3300 cal BCE). A large variety of animal management and hunting strategies can be seen for sites of Phase 1 (Figs 2 and 3), followed by a slight contraction in variation in Phase 2 towards a more restricted focus on the management of sheep and goats (60.0% of domesticates average across all sites compared with 53.1% average in Phase 1). Levels of hunting also increase slightly during this phase relative to phase 1. Phase 2 sites demonstrate an average wild proportion of 5.2% compared with 3.3% average wild representation during Phase 1.

Beginning during the subsequent EB Ib (Phase 3: c.3300-2900 cal BCE), urban sites are first argued to appear in the southern Levant (e.g. [22]; [120]). However, as can be seen in Figs 2 and 3, both rural and putative urban sites continue to demonstrate a range of choices in animal management with no clear pattern in taxonomic proportions between rural and urban groups. Hunting remains of little importance across sites in this phase (3.6% wild) and the focus on the management of sheep and goats continues (70.2% of domesticates). Only two samples were available from this phase for sites identified as potentially urban—Ai/et-Tell and Tel Erani. Although Tel Erani exhibits a less restricted focus on ovicaprines than the average for this period (65.6% compared with 71.2% at rural sites) due to its slightly increased representation of cattle (21.7% compared with 19.3%) as well as pigs (9.8% vs. 7.0%) and equids (2.9% vs. 2.4%), Ai et-Tell does not follow this pattern (13.9% cattle, 0.02% pigs and no equids) and both are well within the overall variation of rural site animal exploitation for this phase (Figs 2 and 3).

Beginning in the EB II (Phase 4: c.2900-2700 cal BCE), multiple sites identified are available, and demonstrate different taxonomic profiles from the remainder of non-urban sites in the southern Levant. Rural sites continue to display little evidence of hunting (1.6% wild) and instead appear to have a focus on the management of sheep and goats (81.9% of domesticates). Sites identified as urban also demonstrate low levels of hunting, albeit higher than those of rural sites (4.3% wild). Within the proportions of domestic animals recovered from these sites, we can additionally see a reduced focus on sheep and goats (58.6% of domesticates) in favour of an increased representation of cattle (36.9% compared with 17.2% at contemporaneous rural sites) as well as a smaller increase in the representation of pigs (4.0% compared with 0.3% at rural sites) and a slightly decreased representation of equids (0.6% compared with 0.8% at rural sites).

The EB III (Phase 5: c.2700-2500 cal BCE) is a continuation of the previous period’s pattern. The differential representation in the presence of cattle between rural and urban sites (29.2% vs. 10.9%) and pigs (2.7% vs. 0.2%) continues from the EB II into the EB III. The representation of equids, however, changes with an increased representation of equids on urban sites (5.8%) relative to rural sites (1.3%). Hunting levels remain very low overall for both rural (2.7%) and urban (6.3%) sites, in keeping with the trend observed in preceding phases. Faunal samples from lower town areas of urban sites, whilst scarce during this (only one sample from this phase) and all periods considered in this study, demonstrate patterns in-between those of rural and urban sites with no pigs present but with 23.0% cattle represented within domesticates and 8.9% of fauna coming from wild taxa (Figs 3 through 4). While not all identified urban sites demonstrate divergent taxonomic distributions, these patterns can be observed at the majority of identified urban sites during this phase. The final period of the Early Bronze Age, the EB IV (Phase 6: c.2500-2000 cal BCE), sees the loss of urban settlement patterns across the southern Levant with the possible exception of Khirbet Iskander or Khirbet al-Batrāwī which demonstrate 3.5% of fauna from wild animals (1.3% from rural sites) but with cattle only representing 8.1% of domesticates (8.2% from rural sites) and pigs only 0.9% compared with 0.5% at rural sites dating to this phase. The similarity of these comparisons does not suggest a difference in diet or access to animal resources between sites of this period, although the limited sample size from the EB IV(where identifiable archaeological sites are less well represented overall) makes this lack of differentiation difficult to prove.

**Fig 4:**
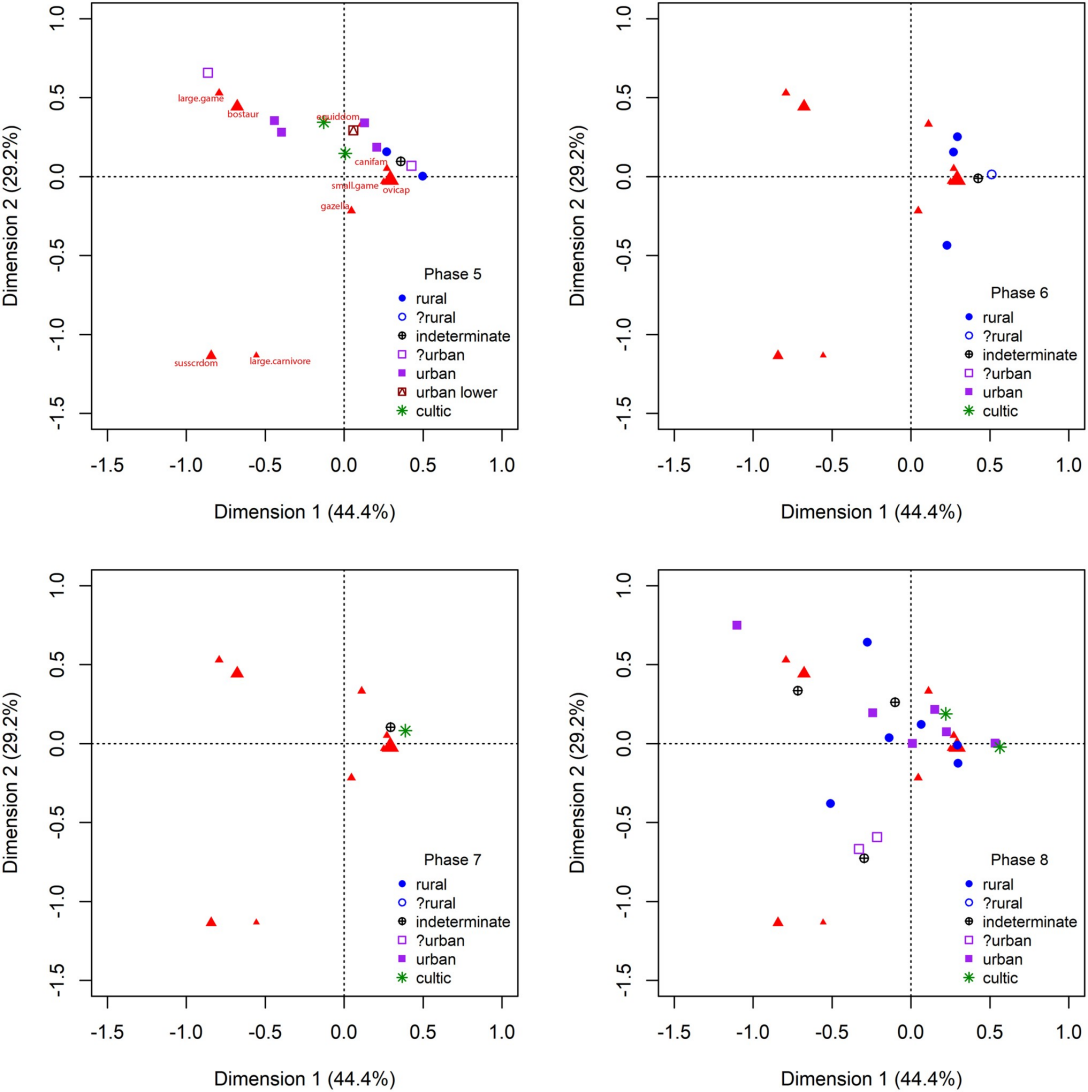
Correspondence analyses of taxonomic distributions for Phases 5 through 8.

As can be seen in Fig 4, there are also very little data available for the first period of the Middle Bronze Age (MB I or Phase 7: c.2000-1900 cal BCE). It is represented only by two samples from Lachish at 1.7% of fauna coming from wild taxa and with domesticates represented by 86.2% sheep and goats, 13.3% cattle and 0.5% pigs. The continuity between the EB IV and MB I patterns may reflect the continuity between the two periods. In fact, it is often difficult to distinguish between them on the basis of the typo-chronological analysis of ceramics, which is why so many archaeologists working in the region combine the two periods and then call the combined period by a neutral term—Intermediate Bronze (IB) ([125]; [42]; [43]; [126]). The return of urbanism can be seen across the southern Levant in the MB II (Phase 8: c.1900-1700 cal BCE). In this phase, two different patterns of urban taxonomic proportions can be identified. Rural sites in this phase continue to demonstrate low levels of hunting (2.6% wild) but with a less restricted focus within domesticates on the management of sheep and goats (65.6% of domesticates) with an increased representation of cattle (21.2%) and pigs (8.0%) compared with periods of the Early Bronze Age. Despite this decrease in rural focus on ovicaprine management, the majority of identified urban sites continue to demonstrate an increased representation of cattle (24.4%) although without an increase in the representation of pigs overall (2.6%) or increase proportions of wild taxa (2.5%), as was seen at urban sites during the preceding EB phases (Phases 4 and 5). Three sites (Tell Jemmeh, Tel Qashish and Yoqne’am), do however continue to demonstrate an increased representation of pigs (26.2% compared), higher than that seen from urban sites in the EB and more similar to that seen at urban sites of Mesopotamia ([127]). These sites are all located along the coastal plain, in areas with comparatively high water availability (in particular Tel Qashish and Yoqne’am). However, other contemporary sites in these same regions do not share similar levels of pig consumption. This is the case even when only urban sites from coastal or well-watered regions are considered. The higher levels of pig consumption at these sites is at present anomalous.

### Ovicaprine age profiles

As discussed above, previous research comparing the age profiles of sheep and goats from urban and rural sites in Mesopotamia and from later periods of the southern Levant demonstrated the exchange of animals from rural ‘producer’ sites to urban ‘consumer’ sites. This can be seen in the truncated age profiles of animals recovered from urban settlements, which exhibit a strong age bias towards the remains of subadult animals. Rural sites, by contrast, demonstrate a corresponding dearth of animals in the subadult age class (e.g. [84]; [81]; [98]; [39], [40]; [103]).

**Fig 5:**
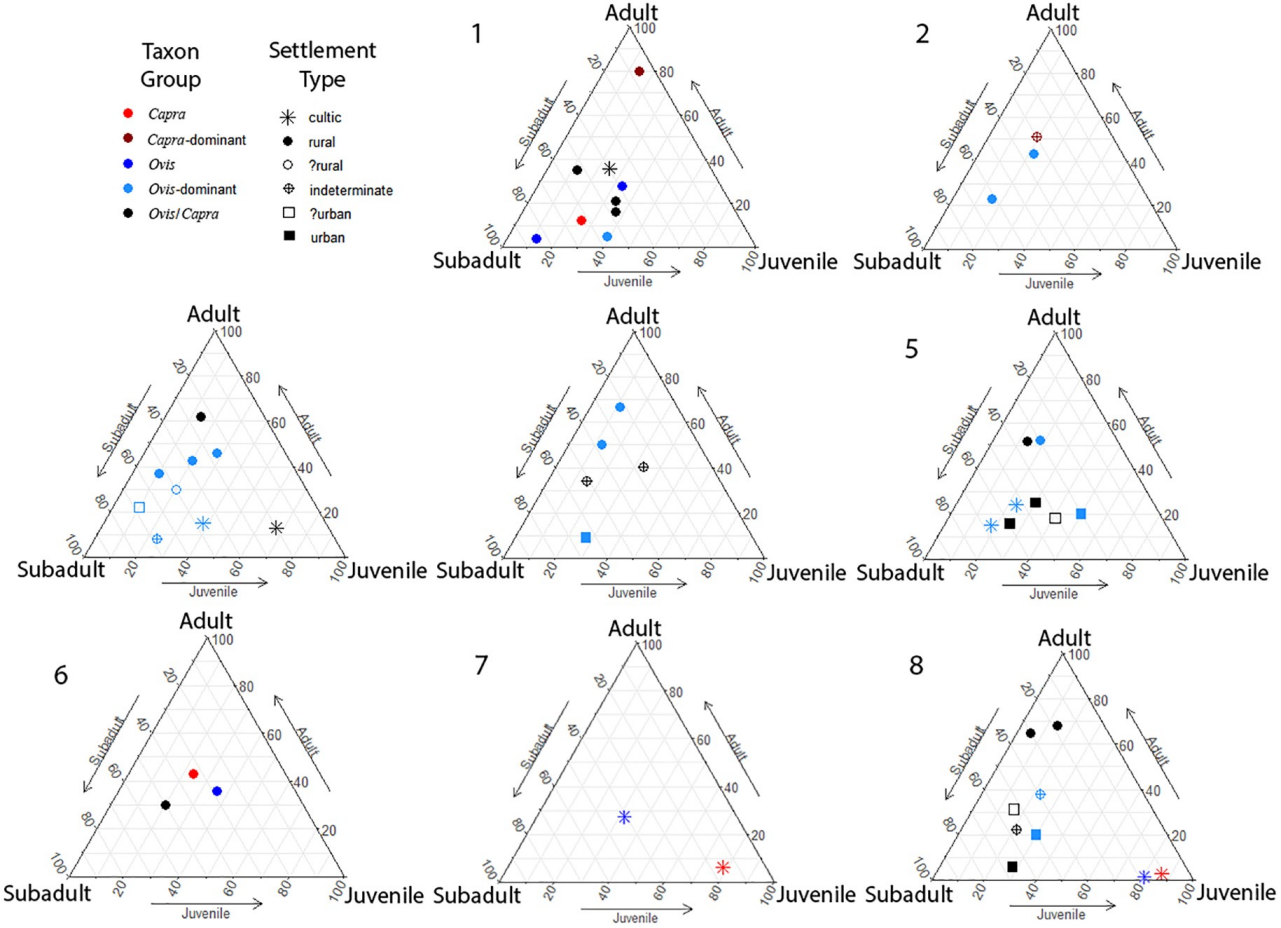
Ternary diagams of dental age profiles for sheep and goats from sites in this study.

In keeping with comparisons of taxonomic proportions, the age profiles from sites pf Phases 1 and 2 (Chalcolithic and EB Ia) demonstrate no overall patterning in the age proportions of sheep and goats (Fig 5). The single putative urban sample from Phase 3 (EB 1b—Ai et-Tell) did not demonstrate discernible differences in taxonomic proportions from rural sites of Phase 3. Comparison of the age profiles for sheep and goats places this single site at the very margins of distributions for this phase, although not clearly differentiation from rural sites in this period. Two additional samples from cultic areas of Megiddo are clearly distinct from the body of rural sites in this phase. These samples together share a higher proportion of subadult animals represented compared with the remainder of ovicaprine age profiles from this phase with the slightly earlier pre-urban EB1b phase at this site. Beginning in the EB II (Phase 4) and continuing and becoming clearly visible in the EB III (Phase 5), we can see a clear pattern of differentiation between rural and urban sites in the age proportions of sheep and goats. These urban sites show a clear bias towards the consumption of subadult animals with a corresponding bias in rural sites towards the consumption of adult animals. This pattern matches that expected for producer vs. consumer sites which is seen from studies of urban sites in Mesopotamia. With the subsequent collapse of urbanism across most (or all) areas of the southern Levant at the end of the EB III this pattern is lost with no putative urban sites against which to compare age profiles for the subsequent EB IV (Phase 6) and MB I (Phase 7). The rural pattern appears to continue as before. In the MB II (Phase 8), the differentiation in age profiles between urban and rural sites is once again found with the return of urbanism.

The divergence of ovicaprine age profiles between rural and urban site groups in both the EB and MB diverges to clearly between these groups to be simply the result of differing patterns of management between sites. The strong biases seen at urban sites towards the consumption of juvenile and subadult animals would have made herds reproductively non-viable (as animals were consumed too young to have enabled the breeding of sufficient numbers to replenish herds) without the supplemental or complete provisioning of sites from elsewhere ([105]; [40]). The confirmation of this pattern as indicating restricted urban-consumer age profiles during the EB II, EB III and MB II is the presence of corresponding contemporaneous rural-producer ovicaprine age profiles with their biases towards adult animals and away from the subadult animals consumed by urban residents ([90]; [105]; [40]).

## Discussion

Even though the EB Ib is the period when sites appear to become extremely large and surrounded by extensive fortification, the limited zooarchaeological data do not show any evidence for divergent patterns of urban provisioning in taxonomic abundance. Zooarchaeological data is available from only two arguably urban sites from this period (Ai et-Tell and Tel Erani) which are not distinguishable in taxonomic composition from the comparatively broad range of animal exploitation practices seen on rural sites in this phase (and preceding phases). The age profile data from Ai et-Tell does, however, indicate differences in provisioning between itself and contemporaneous and rural sites of EB Ib. This small divergence, particularly when compared with patterns of the succeeding EB II and EB III, suggests that the dynamic of rural-producer and urban-consumer sites may have developed during the EB Ib. However, additional comparative faunal data and—in particular—dental age profiles from putative urban sites of this period are necessary in order to determine conclusively whether or not the large fortified settlements developing during the EB Ib demonstrate patterns of urban provisioning. A more finely-grained chronological resolution for sites of the EB1b would be needed to determine if the comparative range of rural site species exploitation practices demonstrates is constant throughout this period or if variation at rural sites constricts towards the later stages of the EB1b towards the more constrained pattern seen in the EB II through MB II.

During the remaining phases of the Early Bronze Age (EB II and EB III, Phases 4 and 5 respectively), urban producer/consumer patterns of provisioning are seen between rural sites and the majority of urban sites in the southern Levant. While some urban sites (Tel Yarmouth, Tel Dalit and Tel Bet Yerah in the EB II and Tel el-Hesi and Tel Yarmouth in the EB III]) do not indicate divergent distributions of taxa during these phases, the majority of identified urban sites from these phases demonstrate a significantly greater proportion of cattle represented within the recovered domesticates in comparison with rural sites. Age profiles for sheep and goats likewise indicate differences in the age distributions of animals consumed at urban vs. rural sites in both periods of putative urbanism in this region. Unlike with the partial divergence between rural and urban site groups seen from taxonomic representation, age profiles show a clear divergence between rural an urban site groups during both the EB II and EB III as well as potentially during the EB Ib at Ai et-Tell. In the MB II (Phase 8) when urban sites are identified in the region, divergent patterns in both domesticate proportions and age profiles are repeated once again.

The majority of urban sites identified from this phase demonstrate an increase in the proportion of cattle compared with that seen at rural sites as well as a bias towards the consumption of subadult rather than adult sheep and goats. This pattern is a duplication of the EB urban patterns. This variation in the representation of cattle is most noticeable at urban sites from areas of comparatively higher rainfall. Cattle have higher water requirements than do sheep and goats (as do pigs), and thus are less well suited to arid regions than their smaller bovid relatives ([109]). Sites located in the driest areas of the southern Levant demonstrate a lower proportion of cattle across the EB and MB in contrast to those of regions with higher precipitation (an average of 10% cattle represented across all sites under 300mm annual precipitation compared with 28.8% across all sites located in regions over 300mm annual precipitation), with a correspondingly lower representation of cattle in urban sites located in areas with annual rainfall under 300mm (see [109]).

Prior to the putative development of urbanism in the EB 1b, sites of the later Chalcolithic and EB1a indicate no overall patterning in the distribution of taxa or in the age distributions of sheep and goats. Within periods of urbanism, clear patterns repeat themselves. While differences rural and urban sites of the EB and MB do not always demonstrate clear differences the distributions of taxa, a clear pattern of differences in the provisioning of sites can be seen from the dental age distributions of sheep and goats. Each of these patterns was subjected to a one-way ANOVA for periods of urbanism (Phases 3 through 8) between site categories and observed variations. While there are some minor differences indicated in the proportions of wild taxa and pigs present recovered from urban sites, in particular during Phases 4 and 5 (Fig 3), neither of these patterns demonstrated a statistically significant variation in either wild (p = 0.202) or pig (0.814) proportions between rural an urban site categories. However, the proportions of cattle represented do show a statistically significant difference in their representation (p = 0.033) between rural and urban site categories. Likewise, dental age distributions show a significant difference (p = 3.03e-7) between urban and rural site categories.

While the increased proportion of cattle across all sites in areas of higher precipitation can be interpreted as resulting from agro-pastoral decisions made regarding the management of livestock in areas with reduced strain on water resources, the higher proportion of cattle specifically at urban sites (compared with rural) cannot be explained merely by the availability of water. Urban (or potentially urban) sites are not restricted to areas of the greatest water availability—other factors must have been involved in the increased consumption of cattle by urban residents. One potential cause for this difference would be the perceived status of cattle as an ‘elite’ food, which would explain its increased consumption on urban sites. Alternatively, cattle may have been kept on urban sites as labour sources in trade activities or for the movement (and storage, e.g. [22]) of agricultural surplus. This may potentially have contributed to the increased representation of cattle during the EB II and III, although one might then expect to see a more consistent increase is also in the presence of other beasts of burden, namely domestic equids (absent during the EB II but present during the EB III although absent again in the MB II). In order to determine the function or status of the increased cattle consumed on urban sites of the EB and MB, comparisons of their age profiles between urban and rural sites would be required, as was done in this study for ovicaprines. Sadly, in the absence of published age information for cattle from the vast majority of either urban or rural sites of the EB and MB the function and/or status of cattle at urban sites of these periods cannot be determined from the available data and must remain for future research.

## Conclusion

When viewed through the lens of a meta-analysis, the zooarchaeological data from the southern Levant can inform us on the nature of early urban and state-level societies in the region and their development. Patterns of differential access to resources are not strongly indicated immediately with the appearance of the earliest large fortified settlements. This challenges the assumption that economic differentiation appears with changes in settlement size ([128], [129]). This economic behaviour can only at present be conclusively identified several hundred years later once these cultural systems have developed regional settlement hierarchies and public institutions (i.e. during the EB II and III). As a result, one cannot assume that unequal access to animal resources will consistently be associated with early state-level societies. Further zooarchaeological work on EB1b sites—rural and urban—with more precise chronological control and involving both age as well as species information is needed to demonstrate rather than assume such a pattern.

Once regional hierarchies and institutions have developed, a pattern of urban vs. rural consumption becomes clearly evident across the region and continues in both the EB and MB. Similarly, urban sites in the southern Levant only demonstrate significantly greater proportions of cattle relative to rural sites at high points of urban state-level development during the EB II, EB III and MB II. The age proportions of sheep and goats on urban sites from both the EB II-III and MB II southern Levant indicate the provisioning of urban ‘consumer’ residents which is distinct from that of from rural ‘producer’ populations. These systematic differences in faunal composition between rural and ‘urban’ sites of the southern Levant across both the EB II-III and MB II strongly indicate separate, urban functions of animal production and consumption.

When taken together with the available evidence for administrative structures, craft production and unequal access to trade, the smaller size of even the largest sites from the southern Levant (in comparison to those from Mesopotamia) does not form a barrier to the consideration of these as urban sites. As the zooarchaeological data suggest, they are engaged in urban-rural relationships with their hinterlands. That this pattern of urban and rural provisioning is repeated across multiple urban sites of the southern Levant during the EB II-III and is resurrected with the return of urban sites in the MB II further supports the identification of these EB sites—though smaller in hectares than their Mesopotamian contemporaries or MB successors—as urban in their nature and function.

These findings confirm the arguments of Cowgill and others ([44]; [119]; [77]; [6]) as to the importance of examining the function of putative urban sites in terms of their embedded relations with their rural hinterlands rather than through a simple checklist of cross-cultural urban attributes such as size and fortifications. The blanket transposition of such checklists from one region onto another (as from Mesopotamia to the southern Levant) is to inaccurately presuppose identical urban form and function between ancient societies rather than to understand the indigenous trajectories of development and networks of settlement in ancient societies. The widespread pattern of urban and rural relationships identified in this study demonstrates that it is this functional relationship embedded within other urban-rural relationships (such as trade and craft production) that serves to identify urban sites. These relationships clearly indicate an urban function for both MB and EB putative urban sites despite the widespread presence of walls at sites of a range of sizes (and functions) and the overall ‘small’ size of EB urban settlements. Additional detailed studies of the local origins of these urban-rural relationships within the southern Levant will further enhance our archaeological understanding of the independent trajectories present in the development of complex societies within this region.

## Supporting information

S1 File Taxonomic database used for correspondence analysis. (CSV)

S2 File Ovicaprine dental age database. (CSV)

S3 File Bibliographic information for zooarchaeological samples included in this study. (DOCX)
