# Supplementary material for: There and back again: A zooarchaeological perspective on Early and Middle Bronze Age urbanism in the southern Levant
Source: PLoS One. 2020 Mar 3;15(3):e0227255. doi: 10.1371/journal.pone.0227255 (PMC7053731; doi:10.1371/journal.pone.0227255)
Supplement: S3 File — (DOCX) [file pone.0227255.s003.docx]

***There and back again: a zooarchaeological perspective on Early and Middle Bronze Age urbanism in the southern Levant***

**Gaastra, J.S., Greenfield, T.L. and Greenfield, H.J.**

**Bibliography for Zooarchaeological Database (Table 2, SI1 and SI2)**

Agha N. Faunal remains from the Early Bronze Age site of Qiryat Ata – Area N. *‘Atiqot* 2014;79: 45-57.

Alhaique F. Faunal remains. In: Nigro L, editor. *Khirbet al-Batrawy II: the EB II City-Gate, the EB II-III Fortifications, the EB II-III Temple. Preliminary Report of the Second (2006) and Third (2007) Seasons of Excavation.* Rome: La Sapienza; 2008. pp. 327-358.

Alhaique F. Faunal remains. In: Nigro L, editor. *Khirbet al-Batrawy III: the EB II-III Triple Fortification Line and the EB IIIb Quarter Inside the City Wall. Preliminary Report on the Fourth (2008) and Fifth (2009) Seasons of Excavations*. Rome: La Sapienza; 2012. pp. 333-364.

Allentuck A. *Human-Livestock Relations in the Early Bronze Age of the Southern Levant*. PhD Thesis, University of Toronto. 2013.

Al-Zawahra M. The faunal remains from Tell el-Mafjer, a Chalcolithic site in the Lower Jordan Valley, Palestine. In: Choyke AM and Buitenhuis H, editors. *Archaeozoology of the Near East VIII, Tome 2*. Rhône-Alpes: Archéorient, Maison de l’Orient et de la Mediterranée; 2008. pp. 431-450.

Bangsgaard P. Animal bones. In: Strand J, editor. *Tall al-Fukhar: Results from Excavations in 1990-90 and 2002. Volume I: Text*. Aarhus: Aarhus University Press; 2015. pp. 378-396.

Berger A. Feeding cities? – Preliminary notes on the provisioning of animal products at Tel Bet Yerah. In: Çakirlar C, Chahod J, Berthon R and Pilaar-Birch, S, editors. *Archaeozoology of the Near East XII. Proceedings of the 12^th^ International Symposium of the ICAZ Archaeozoology of Southwest Asia and Adjacent Areas Working Group, Groningen Institute of Archaeology, June 14-15 2015, University of Groningen, Netherlands*. Groningen: Barkhuis Publishing; 2018. pp. 13-25.

Bourke SJ, Lovell JL, Sparks RT, Seaton PL, Mairs L and Meadows J. A second and third season of renewed excavations by the University of Sydney at Tulaylāt al-Ghassūl. *Annual of the Department of Antiquities of Jordan* 2000;44: 37-89.

Bourke SJ, Seaton PL, Sparks RT, Lovell JL and Mairs LD. A first season of renewed excavations by the University of Sydney at Tulaylāt al-Ghassūl. *Annual of the Department of Antiquities of Jordan* 1995;39: 31-63.

Bourke SJ, Sparks RT, Sowada KN and Mairs LD. Preliminary report on the University of Sydney’s fourteenth season of excavations at Pella (Tabaqat Faḥl) in 1992. *Annual of the Department of Antiquities of Jordan* 1994;38: 81-126.

Bourke SJ, Sparks RT, Sowada KN, McClaren PB and Mairs LD. Preliminary report on the University of Sydney’s sixteenth and seventeenth excavations at Pella (Tabaqat Faḥl) in 1994/95. *Annual of the Department of Antiquities of Jordan* 1998;42: 179-211.

Cope CR. The fauna: preliminary results. In: Geztov N, editor. *The Tel Bet Yeraḥ Excavations, 1994-1995. Israel Antiquities Authority Reports 28.* Jerusalem: Israel Antiquities Authority; 2006. pp. 169-174.

Croft P. The osteological remains (mammalian and avian). In: Ussishkin D, editor. *The Renewed Archaeological Investigations at Lachish (1973-1994)*. Tel Aviv: Emery and Claire Yass Publications in Archaeology; 2004. pp. 2254-2452.

Davis SJM. The mamma bones: Tel Yarmouth 1980-1983. In: de Miroschedji P, editor. Yarmouth I: Rapport sur les Trois Premièrs Campagnes de Fouilles á Tel Yarmouth (Israël) (1980-1982). Paris: Éditions Recherche sur les Civilisations; 1988. pp. 143-149.

Ducos P. *L’Origine des Animaux Domestiques en Palestine*. Bordeaux: Imprimeries Delmas. 1968.

Finnegan M. Faunal remains from Bab edh-Dhra and Numeira. In: Rast WE and Schaub RT, editors. *The Southeastern Dead Sea Plain Expedition: an Interim Report of the 1977 Season*. Cambridge, Massachusetts: American Schools of Oriental Research; 1979. pp. 177-180.

Fischer PM. Tall Abū al-Kharaz: occupation throughout the ages. The faunal and botanical evidence. *Studies in the History and Archaeology of Jordan* 1997;6: 159-165.

Gibbs K, Allentuck A, Kadowaki S and Banning EB. Early Bronze 1 occupation at al-Basatîn, in Wadi Ziqlab, northern Jordan. *Bulletin of the American Schools of Oriental Research* 2009;355: 31-50.

Golani A. The Early Bronze Age site of Ashquelon Afridar – Area M. *‘Atiqot* 2008;60: 19-51.

Golani A, van den Brink ECM, Goren Y, Marder O, Rowan Y, Shamir O, Bar-Yosef Mayer DE, Boas A. and Horwitz LK. Salvage Excavations at the Early Bronze Age Ia settlement of Azor. *‘Atiqot* 1999;38: 1-49.

Grigson C. The mammalian remains from the Chalcolithic site of Ḥorvat Beter: excavations of 1982. *‘Atiqot* 1993;22: 28-31.

Grigson C. Cattle keepers of the northern Negev: animal remains from the Chalcolithic site of Grar. In: Gilead I, editor. *Grar: a Chalcolithic Site in the Northern Negev.* Tel Aviv: Ben Gourion University of the Negev Press; 1995. pp. 377-452.

Grigson C. Farming? Feasting? Herding? Large mammals from the Chalcolithic of Gilat, Israel. In: Levy TE, editor. *Archaeology, Anthropology and Cult: the Sanctuary at Gilat, Israel*. London: Equinox; 2006. pp. 215-319.

Griffith TB. *Ancient Economic Complexity and Adaptation in the Semi-Arid Highlands of Jordan: a Zooarchaeological Study of the Early Bronze Age Through the Ottoman Period at Tell Madaba*. PhD Thesis, University of Kansas. 2016.

Hellwing S. Animal bones from Tel Tsaf. *Tel Aviv* 1988;15(1): 47-51.

Hellwing S. Faunal remains from the Early and Late Bronze Ages at Tel Kinrot. *Tel Aviv* 1989;16(2): 212-220.

Hellwing, S. Faunal Remains. In: Kochavi M, Beck P and Yadin E, editors. *Aphek-Antipatris I. Excavations of Areas A and B: the 1972-1976 Seasons.* Monograph Series 19, Institute of Archaeology, Tel Aviv University. Tel Aviv: Institute of Archaeology, Tel Aviv University; 2000. pp. 293-314.

Hellwing S, Sade M and Kishon V. Faunal remains. In: Finkelstein I, Bunimovitz S and Lederman Z, editors. *Shiloh: the Archaeology of a Biblical Site*. Tel Aviv: Monograph Series of the Institute of Archaeology; 1993 pp. 309-367.

Hesse B and Wapnish P. Commodities and cuisine: animals in the Early Bronze Age of northern Palestine. In: Wolff SR, editor. *Studies in the Archaeology of Israel and Neighbouring Lands in Memory of Douglas L. Esse*. Atlanta: American School of Oriental Research ASOR Books; 2001. pp. 251-305.

Hill AC. Specialized Pastoralism and Social Stratification – Analysis of the Fauna from Chalcolithic Tel Tsaf, Israel. PhD Thesis, University of Connecticut. 2011.

Hill AC, Price MD and Rowan YM. Feasting at Marj Rabba, an early Chalcolithic site in the Galilee. *Oxford Journal of Archaeology* 2016;35(2): 127-140.

Horwitz LK. Sedentism in the Early Bronze IV: a faunal perspective. *Bulletin of the American Schools of Oriental Research* 1989;275: 15-25.

Horwitz LK. Faunal remains. In: Braun E, editor. *Yiftaḥ’el: Salvage and Rescue Excavations at a Prehistoric Village in Lower Galillee, Israel. Israel Antiquities Authority Reports 2*. Jerusalem: Israel Antiquities Authority; 1997. pp. 155-171.

Horwitz LK. The faunal remains. In: Edelstein G, editor. *Villages, Terraces and Stone Mounds: Excavations at Manaḥat, Jerusalem, 1987-1989*. Israel Antiquities Authority Reports No. 3. Jerusalem: Israel Antiquities Authority; 1998. pp. 104-112.

Horwitz LK. Animal Bones. In: Kempinski A, editor. *Tel Kabri: the 1986-1993 Excavation Seasons*. Tel Aviv: Emery and Claire Yass Publications in Archaeology; 2002. pp. 395-401.

Horwitz LK. Early Bronze Age animal exploitation at Qiryat Ata. In: Golani A, editor. *Salvage Excavations at the Early Bronze Age Site of Qiryat Ata. Israel Antiquities Authority Reports 18*. Jerusalem: Israel Antiquities Authority; 2003a. pp. 225-241.

Horwitz LK. Fauna from Tel Qashish. In: Ben-Tor A, Bonfil R and Zuckerman S, editors. *Tel Qashish: a Village in the Jezreel Valley. Final Report on the Archaeological Excavations 1978-1987. Qedem Reports 5*. Jerusalem: Institute of Archaeology, Hebrew University of Jerusalem; 2003b. pp. 427-443.

Horwitz LK. Early Bronze Age fauna from Qiryat Ata – Area O. *‘Atiqot* 2013;75: 61-70.

Horwitz LK, Giora NB, Mienis HK and Lernau O. Faunal and malacological remains from the Middle Bronze, late Bronze and Iron Age levels at Tell Yoqne'am. In: Ben-Tor A, Men-Ami D and Livneh A, editors. *Yoqne'am III: the Middle and Late Bronze Ages. Final Report of the Archaeological Excavations (1977-1988). Quedem Reports 7*. Jerusalem: Israel Exploration Society and the Hebrew University of Jerusalem; 2005. pp.393-435.

Horwitz LK, Hellwing S and Tchernov E. Patterns of animal exploitation. In: Gophna R, editor. *Excavations at Tel Dalit*. Tel Aviv: Ramot Publishing; 1996. pp. 193-216.

Josien T. La faune Chalcolithique des Gisements Palestiniens de Bir-es Safadi et Bir Abou Matar. *Israel Exploration Journal* 1955;5(4): 246-256.

Kansa, EC, Kansa, SW and Levy, T. Eat like an Egyptian? – A contextual approach to an Early Bronze I “Egyptian colony” in the southern Levant. In: Maltby, M, editor, *Integrating Zooarchaeology*. Oxford: Oxbow Books; 2006. p. 76-91.

Kansa SW. Animal Exploitation at Early Bronze Age Ashqelon, Afridar: What the Bones Tell Us—Initial Analysis of the Animal Bones from Areas E, F and G*. 'Atiqot* 2004;45: 279-297.

Klenck JD. *Animals in the Canaanite Cultic Milieu: the Zooarchaeological Evidence from Tel Haror, Israel.* PhD Thesis, University of Michigan. 1996.

Klenck, J.D. *The Canaanite Cultic Milieu: the Zooarchaeological Evidence from Tel Haror, Israel. BAR International Series 1029*. Oxford: Archaeopress; 2002.

Lernau H. Faunal remains, strata III-I. In: Amiran R, editor. *Early Arad: the Chalcolithic Settlement and Early Bronze City I: First-Fifth Seasons of Excavations 1962-1966*. Jerusalem: Israel Exploration Society; 1978. pp. 83-113

Maher EF. Animal-based economy and local ecology: the Early Bronze Age II fauna from Qiryat Ata – Area S. *‘Atiqot* 2014a;79: 99-109.

Maher EF. Temporal trends in animal exploitation: faunal analysis from Tell Jemmeh. In: Ben-Schlomo D and van Beek GW, editors. *The Smithsonian Institution Excavation at Tell Jemmeh, Israel, 1970-1990*. Washington, DC: Smithsonian Institution Scholarly Press; 2014b. pp. 1038-1053.

Makarewicz CA. Pastoral production in a corporate system: the Early Bronze Age at Khirbet el-Minsahlat, Jordan. In: Buitenhuis H, Choyke AM, Martin L, Bartosiewicz L and Mashkour M, editors. *Archaeozoology of the Near East VI: Proceedings of the Sixth International Symposium on the Archaeozoology of Southwestern Asia and Adjacent Areas*. Groningen: ARC Publicaties; 2005. pp. 163-177.

Marom N, Lev-Tov J and Kehati R. Zooarchaeological reports. In: Ben-Tor A, Zuckerman S, Bechar S and Sandhaus D, editors. *Hazor VII: the 1990-2012 Excavations: The Bronze Age*. Jerusalem: Israel Exploration Society; 2017. pp. 661-699.

Marom N, Yasur-Landau A and Cline EH. The silent coast: zooarchaeological evidence to the development trajectory of a second millennium palace at Tel Kabri. *Journal of Anthropological Archaeology* 2015;39: 181-192.

Metzger MC. Faunal remains from Area C. In: Richard S, Long Jr. JC, Holdorf PS and Peterman G, editors. *Khirbat Iskander: Final Report on the Early Bronze IV Area C “Gateway” and Cemeteries*. Boston, Massachusetts: American Schools of Oriental Research; 2010. pp. 141-143.

Peck-Janssen SM. *Animal Husbandry at Tell el Hesi (Israel): Results from Zooarchaeological and Isotopic Analysis*. MA Thesis, University of South Florida. 2006.

Peters J, Pöllath N and von den Driesch A. Early and Late Bronze Age transitional subsistence at Tall al-‘Umayri. In: Herr LG, Clark DR, Geraty LT, Younker RW and LaBianca ØS, editors. *Madaba Plains Project: The 1994 Season at Tall al-‘Umayri and Subsequent Studies*. London: Andrews University Pres; 2002. pp. 305-347.

Price MD, Buckley M, Kersel MM and Rowan YM. Animal management strategies during the Chalcolithic in the lower Galilee: new data from Marj Rabba (Israel). *Paléorient* 2013;39(2): 183-200.

Sade M. Animal bones from Qiryat Ata – Area L. *Tel Aviv* 2000;27: 57-60.

Sade M. Archaeozoological finds from the excavations west of Tel Qasile. *‘Atiqot* 2006;53: 135-137.

Sade M. The archaeozoological remains. In: Greenhut Z and De Groot A, editors. *Salvage Excavations at Tel Moza: The Bronze and Iron Age Settlements and Later Occupations*. Israel Antiquities Authority Reports 39. Jerusalem: Israel Antiquities Authority; 2009. pp. 197-207

Seger JD, Baum B, Borowski O, Cole DP, Forshey H, Futato E, Jacobs PF, Laustrup M, O’Connor Seger P and Zeder M. The Bronze Age settlements at Tel Halif: phase II excavations 1983-1987. *Bulletin of the American Schools of Oriental Research, Supplementary Studies* 1990;26: 1-32.

van den Brink ECM, Horwitz LK, Kool R, Liphschitz N, Mienis HK and Zbenovich V.Excavations at Tel Lod: remains from the Pottery Neolithic A, Chalcolithic, Early Bronze Age I, Middle Bronze Age I and Byzantine periods. ‘*Atiqot* 2015;82: 141-218.

van den Brink ECM, Schmueli O, Yannai E, Horwitz LK and Vadaei E. Middle Bronze Age IIa and later settlement remains near Yehud on the coastal plain. *‘Atiqot* 2014;79: 131-174.

Wapnish P and Hesse B. Urbanization and the organization of animal production at Tell Jemmeh in the Middle Bronze Age Levant. *Journal of Near Eastern Studies* 1988;47(2): 81-94.

Wapnish P and Hesse B. Faunal remains from Tel Dan: perspectives on animal production at a village, urban and ritual center. *Archaeozoologia* 1991;4(2): 9-86.

Wapnish P. and Hesse B. 2001. Mammal remains from the Early Bronze sacred compound. In: Finkelstein I, Ussishkin D and Halpern B, editors. *Megiddo III: the 1992-1996 seasons*. Tel Aviv: Emery and Claire Yass Publications in Archaeology; 2001. pp. 429-477.

Wichiter S. *Animals, Environment and Society: a Zooarchaeological Approach to the Late Chalcolithic – Early Bronze I Transition in the Southern Levant*. PhD Thesis, University of Edinburgh. 1999.

Wojtal P, Wertz K, Tomek T, Lõugas L and Żychowska E. Tel Erani faunal remains. In: Ciałowicz, KM, Yekutieli, Y and Czarnowicz, M, editors, *Tel Erani I: Preliminary Report of the 2013-2015 Excavations*. Krakow: Wydawnictwo Alter; 2016. pp.117-125.
